# Pediatric Sepsis Research: Where Are We and Where Are We Going?

**DOI:** 10.3389/fped.2022.829119

**Published:** 2022-02-11

**Authors:** Letícia Massaud-Ribeiro, Pedro Henrique Nunes Costa Silami, Fernanda Lima-Setta, Arnaldo Prata-Barbosa

**Affiliations:** ^1^Pediatric Intensive Care Unit, Department of Pediatrics, Instituto de Puericultura e Pediatria Martagão Gesteira, Universidade Federal do Rio de Janeiro, Rio de Janeiro, Brazil; ^2^Pediatric Intensive Care Unit, Department of Pediatrics, Instituto Fernandes Figueira, Fundação Oswaldo Cruz, Rio de Janeiro, Brazil; ^3^Pediatric Intensive Care Unit, Department of Pediatrics, Hospital Estadual da Criança, Rio de Janeiro, Brazil; ^4^Department of Pediatrics, Instituto D'Or de Pesquisa e Ensino, Rio de Janeiro, Brazil

**Keywords:** sepsis, septic shock, pediatrics, pediatric intensive care, bibliometrics

## Abstract

Sepsis continues to be one of the leading causes of admission to the Pediatric Intensive Care Unit, representing a great challenge for researchers and healthcare staff. This mini review aims to assess research on pediatric sepsis over the years. Of the 2,698 articles retrieved from the Scopus database, the 100 most cited were selected (50 published since 2000 and 50 published since 2016). The most cited studies, published in the 21st century, are highlighted, with their main findings and perspectives.

## Introduction

Global estimates of sepsis burden in children reveal an incidence of 1.2 million cases per year, with mortality ranging from 1 to 5% for sepsis and 9–20% for severe sepsis (1, 2). Younger age, unknown or incomplete vaccination status, non-adherence to sepsis treatment bundles, healthcare-associated infection, underlying cardiovascular condition, and multiple organ dysfunction are factors that may be associated with higher odds of mortality (2, 3). Although entirely plausible, further studies are needed to validate them in a broader and more robust way. A 2019 systematic review demonstrated a declining trend of case-fatality rates in severe pediatric sepsis and septic shock. However, significant disparities exist between low- and middle-income countries (LMIC) vs. high-income countries (4). In South America, a 2021 study showed a high prevalence of sepsis and sepsis-related mortality in a sample of children admitted to Pediatric Intensive Care Unit (PICU), with a quarter of deaths occurring within the first 24 h of PICU admission (2).

In addition to being a significant cause of PICU admission and mortality, sepsis also accounts for the high consumption of health resources. A cohort study of severe pediatric sepsis in the United States showed a median length of stay (LOS) of 7 days in the PICU and 17 days in the hospital, accounting for a median total hospitalization cost of 77,446 USD per admission (3). Disability is also an important cause of the sepsis burden, even in high-income countries. A prospective cohort study in Europe showed that nearly one-third of community-acquired sepsis survivors admitted to the PICU were discharged with some disability, including 24% of previously healthy children who survived with disability (5).

Most epidemiologic studies and clinical trials with septic children have been conducted in high-income countries (1, 6–10). Epidemiologic data on pediatric sepsis is particularly needed in LMIC, as infectious diseases account for an important part of life-threatening conditions in children in these areas (11). The availability of these data is essential for elaborating public healthcare policies, allowing for more rational allocation of limited financial resources and soothing social and economic disparities in childhood mortality.

The current state of pediatric diagnosis and management is driven by the most highly cited manuscripts in the literature. Understanding the quality of the evidence may inform potential future directions for research and potentially clinical practice. To be able to address this, we first hypothesize that characterizing the nature (definitions, diagnosis, management) and quality of evidence for the manuscripts that have the highest number of citations will inform gaps in knowledge as they are currently the most widely used articles in the field.

## Methods

We chose to search the Scopus database, a multidisciplinary database with the greatest coverage of journals in the healthcare field and which present the number of citations per article. It was accessed on September 14th, 2021. No article type limit was set for the search, and keywords were searched only in the title field. The search query was the following: *[TITLE (“Sepsis” OR “Severe Sepsis” OR “Septicemia” OR “Septicemias” OR “Sepsis Syndrome” OR “Sepsis Syndromes” OR “septic shock”) AND TITLE (“child” OR “children” OR “pediatric” OR “pediatric” OR “pediatrics” OR “pediatrics” OR “infant” OR “infants”) AND NOT TITLE (“newborn” OR “newborns” OR “neonate” OR “neonates” OR “preterm” OR “preterms” OR “premature” OR “prematures” OR “very-low-birth-weight” OR “low-birth-weight”)]*. The retrieved articles were analyzed by the Scopus algorithm and classified by the following characteristics: affiliations, article type, authors, country, funding sponsor, journal, and year of publication. Articles were also ranked by the number of citations.

From a statistical point of view, our sample was a convenience sample. Due to space limitations and the scope of this mini review, we decided to select the 100 most cited articles. However, the older the articles, the greater the possibility of citation. So, we adopted an additional criterion: selecting 50 articles representing publications since the beginning of the century (2000–2021) and 50 representing more recent years (2016–2021), excluding the duplicates. Articles addressing neonatal sepsis were also excluded. The remaining articles were analyzed for their relevance, initially by title, and in case of any ambiguity, by their abstracts and full texts, when necessary.

## Results

**Figure 1:**
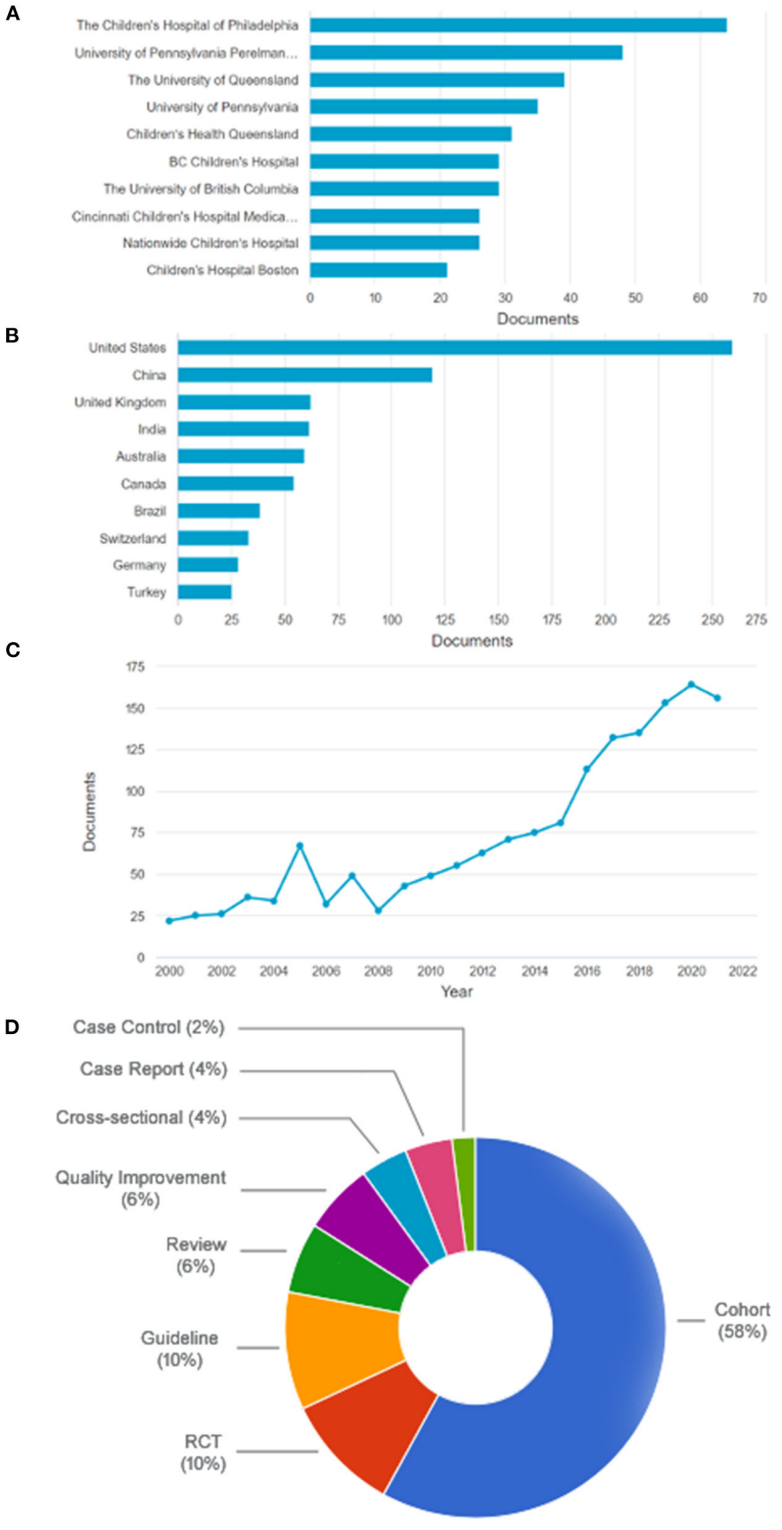
**(A)** The ten most frequent institutions among the most cited articles in the last 5 years (all from North America or Australia). **(B)** The ten most frequent countries among the most cited articles in the last 5 years. **(C)** All articles retrieved sorted by year. **(D)** Types of articles. RCT, randomized controlled trial. Based on data from Scopus website.

The electronic search yielded 2,698 articles. All ten institutions whose authors published most frequently in the last 5 years are located in North America or Australia (Figure 1A). Considering the country of origin, middle-income countries, such as India, China, Brazil, Turkey, and others, account for 29.2% of publications (Figure 1B). There has been a worldwide steady increase in pediatric sepsis publications over the years (Figure 1C). Despite this recent increase in published studies, a 2021 meta-analysis reported that LMIC countries account for only about 20% of the included studies and 2% of the total patient sample (12). China, India, and Brazil are heavily populated continental countries with significant social inequalities. China alone has been among the top ten funding sponsors from these three emerging countries over the past 5 years. Brazil has been making systematic cuts in the public research budget in recent years (13).

Of the 100 most cited, 12 were excluded for duplicity or addressing neonatal sepsis. The 50 most cited articles in both studied periods are shown in Table 1. Most have a low level or very low level of evidence (68 and 14%, respectively), with only 12% with a moderate level and 6% with a high level of evidence, according to the GRADE system (www.gradeworkinggroup.org) (Figure 1D).

**Table 1:** The 50 most cited articles retrieved since 2,000 in Scopus database, ranked by the number of citations.

**#**	**First author**	**Article title**	**Journal**	**Year**	**Country**	**Type of study**	**Level of evidence^a^**	**Citations**
1	Goldstein B	International pediatric sepsis consensus conference: definitions for sepsis and organ dysfunction in pediatrics.	*Pediatr Crit Care Med*	2005	USA	Guidelines	Very low	2,027
2	Brierley J	Clinical practice parameters for hemodynamic support of pediatric and neonatal septic shock: 2007 update from the ACCM.	*Crit Care Med*	2009	USA	Guidelines^c^	Low	706
3	Watson RS	The epidemiology of severe sepsis in children in the United States.	*Am J Respir CCM*	2003	USA	Cohort	Low	583
4	Carcillo JA	Clinical practice parameters for hemodynamic support of pediatric and neonatal patients in septic shock.	*Crit Care Med*	2002	USA	Guidelines^c^	Low	486
5	Han YY	Early reversal of pediatric-neonatal septic shock by community physicians is associated with improved outcome.	*Pediatrics*	2003	USA	Cohort	Low	418
6	Nadel S	Drotrecogin alfa (activated) in children with severe sepsis: a multicentre phase III randomized controlled trial.	*Lancet*	2007	USA^b^	RCT	High	396
7	Weiss SL	Global epidemiology of pediatric severe sepsis: the sepsis prevalence, outcomes, and therapies study.	*Am J Respir CCM*	2015	USA^b^	Cross-sectional	Low	384
8	Levin M	Recombinant bactericidal/permeability-increasing protein (rBPI21) as adjunctive treatment for children with severe meningococcal sepsis: a randomized trial.	*Lancet*	2000	USA^b^	RCT	High	337
9	Hartman ME	Trends in the epidemiology of pediatric severe sepsis.	*Pediatr Crit Care Med*	2013	USA	Cohort	Low	318
10	Oliveira CF	ACCM/PALS haemodynamic support guidelines for pediatric septic shock: an outcomes comparison with and without monitoring central venous oxygen saturation.	*Intensive Care Med*	2008	Brazil	RCT	Moderate	281
11	Wheeler DS	Serum neutrophil gelatinase-associated lipocalin (NGAL) as a marker of acute kidney injury in critically ill children with septic shock.	*Crit Care Med*	2008	USA	Cohort	Moderate	269
12	Davis AL	ACCM clinical practice parameters for hemodynamic support of pediatric and neonatal septic shock.	*Crit Care Med*	2017	USA	Guidelines^c^	Low	250
13	Fleischmann-Struzek C	The global burden of pediatric and neonatal sepsis: a systematic review.	*Lancet Respir Med*	2018	Canada^b^	Systematic review	Moderate	244
14	Panigrahi P	A randomized synbiotic trial to prevent sepsis among infants in rural India.	*Nature*	2017	USA	RCT	Moderate	236
15	Adem P V	Staphylococcus aureus sepsis and the Waterhouse-Friderichsen syndrome in children.	*N Engl J Med*	2005	USA	Case report	Very low	231
16	De Kleijn ED	Activation of protein C following infusion of protein C concentrate in children with severe meningococcal sepsis and purpura fulminans: a randomized, double-blinded, placebo-controlled, dose-finding study.	*Crit Care Med*	2003	USA^b^	RCT	High	210
17	Weiss SL	Delayed antimicrobial therapy increases mortality and organ dysfunction duration in pediatric sepsis.	*Crit Care Med*	2014	USA	Cohort	Low	203
18	Felmet KA	Prolonged lymphopenia, lymphoid depletion, and hypoprolactinemia in children with nosocomial sepsis and multiple organ failure.	*J Immunol*	2005	USA	Cohort	Low	200
19	Watson RS	Scope and epidemiology of pediatric sepsis.	*Pediatr Crit Care Med*	2005	USA	Review	Low	178
20	Wong HR	Genome-level expression profiles in pediatric septic shock indicate a role for altered zinc homeostasis in poor outcome.	*Physiol Genomics*	2007	USA	Case-control	Very low	176
21	Matics TJ	Adaptation and validation of a pediatric sequential organ failure assessment score and evaluation of the Sepsis-3 definitions in critically ill children.	*JAMA Pediatr*	2017	USA	Cohort, validation	Low	170
22	Ruth A	Pediatric severe sepsis: current trends and outcomes from the pediatric health information systems database.	*Pediatr Crit Care Med*	2014	USA	Cohort	Low	168
23	Schlapbach LJ	Mortality related to invasive infections, sepsis, and septic shock in critically ill children in Australia and New Zealand, 2002–13: a multicentre retrospective cohort study.	*Lancet Infect Dis*	2015	Australia, New Zealand	Cohort	Moderate	163
24	Weiss SL	Surviving sepsis campaign international guidelines for the management of septic shock and sepsis-associated organ dysfunction in children.	*Pediatr Crit Care Med*	2020	USA, Europe^b^	Guidelines^c^	Low	156
25	Odetola FO	Patient and hospital correlates of clinical outcomes and resource utilization in severe pediatric sepsis.	*Pediatrics*	2007	USA	Cohort	Low	156
26	Balamuth F	Pediatric severe sepsis in U.S. children's hospitals.	*Pediatr Crit Care Med*	2014	USA	Cohort	Low	155
27	Wong HR	Developing a clinically feasible personalized medicine approach to pediatric septic shock.	*Am J Respir CCM*	2015	USA	Cohort, validation	Low	150
28	Pizarro CF	Absolute and relative adrenal insufficiency in children with septic shock.	*Crit Care Med*	2005	Brazil, USA	Cohort	Low	146
29	Cruz AT	Implementation of goal-directed therapy for children with suspected sepsis in the emergency department.	*Pediatrics*	2011	USA	Quality improvement	Very low	145
30	Wong HR	Genomic expression profiling across the pediatric systemic inflammatory response syndrome, sepsis, and septic shock spectrum.	*Crit Care Med*	2009	USA	Cohort	Low	145
31	Blomberg B	High rate of fatal cases of pediatric septicemia caused by gram-negative bacteria with extended-spectrum beta-lactamases in Dar es Salaam, Tanzania.	*J Clin Microbiol*	2005	Tanzania	Cohort	Low	144
32	Hatherill M	Procalcitonin and cytokine levels: relationship to organ failure and mortality in pediatric septic shock.	*Crit Care Med*	2000	UK	Cohort	Low	141
33	Wong HR	Identification of pediatric septic shock subclasses based on genome-wide expression profiling.	*BMC Medicine*	2009	USA	Cohort	Moderate	140
34	Larsen GY	An emergency department septic shock protocol and care guideline for children initiated at triage.	*Pediatrics*	2011	USA	Quality improvement	Low	138
35	Harvala H	Specific association of human parechovirus type 3 with sepsis and fever in young infants, as identified by direct typing of cerebrospinal fluid samples.	*J Infect Dis*	2009	UK	Cross-sectional	Low	138
36	Inwald DP	Emergency management of children with severe sepsis in the united kingdom: the results of the pediatric intensive care society sepsis audit.	*Arch Dis Child*	2009	UK	Cohort	Low	132
37	Standage SW	Biomarkers for pediatric sepsis and septic shock.	*Expert Rev Anti Infect Ther*	2011	USA	Review	Very low	130
38	MacLaren G	Central extracorporeal membrane oxygenation for refractory pediatric septic shock.	*Pediatr Crit Care Med*	2011	Australia	Case report	Very low	129
39	Carcillo JA	Cytochrome P450 mediated-drug metabolism is reduced in children with sepsis-induced multiple organ failure.	*Intensive Care Med*	2003	USA	Cohort	Low	122
40	Branco RG	Glucose level and risk of mortality in pediatric septic shock.	*Pediatr Crit Care Med*	2005	Brazil, UK	Cohort	Low	121
41	Mickiewicz B	Metabolomics as a novel approach for early diagnosis of pediatric septic shock and its mortality.	*Am J Respir CCM*	2013	Canada	Cohort	Low	117
42	Wong HR	The pediatric sepsis biomarker risk model	*Crit Care*	2012	USA	Cohort	Low	114
43	Kutko MC	Mortality rates in pediatric septic shock with and without multiple organ system failure.	*Pediatr Crit Care Med*	2003	USA	Cohort	Low	114
44	Oliveira CF	Time- and fluid-sensitive resuscitation for hemodynamic support of children in septic shock: barriers to the implementation of the ACCM/PALS in a PICU in a developing world.	*Pediatr Emerg Care*	2008	Brazil, USA	Cohort	Low	112
45	Joosten KF	Endocrine and metabolic responses in children with meningococcal sepsis: striking differences between survivors and non-survivors.	*J Clin Endocrinol Metab*	2000	Netherlands	Cohort	Low	111
46	Nguyen TC	Acquired ADAMTS-13 deficiency in pediatric patients with severe sepsis.	*Haematologica*	2007	USA	Cohort	Low	110
47	Wong HR	Interleukin-8 as a stratification tool for interventional trials involving pediatric septic shock.	*Am J Respir CCM*	2008	USA	Cohort	Low	108
48	Den Brinker M	One single dose of etomidate negatively influences adrenocortical performance for at least 24 h in children with meningococcal sepsis.	*Intensive Care Med*	2008	Netherlands	Cohort	Low	108
49	Demirkol D	Hyperferritinemia in the critically ill child with secondary hemophagocytic lymphohistiocytosis/sepsis/multiple organ dysfunction syndrome/macrophage activation syndrome: what is the treatment?	*Crit Care*	2012	Turkey	Cohort	Low	106
50	Kissoon N	World federation of pediatric intensive care and critical care societies: global sepsis initiative.	*Pediatr Crit Care Med*	2011	^*^	Quality improvement	Very low	106

a *Based on the GRADE Working Group recommendations;*

b *Based on coordinating center or senior author affiliation;*

c *Based on most studies used in consensus*.

* *Canada, USA, South Africa, France, India, and The Netherlands*.

## Discussion

### Sepsis Definitions

The most cited article of the 21st century was the International Pediatric Sepsis Consensus Conference (IPSCC) on definitions for sepsis and organ dysfunction in Pediatrics (14). This critical study has been used as a reference for research in pediatric sepsis to this day. It defined a systemic inflammatory response syndrome (SIRS) based on age-related abnormal body temperature, heart rate, respiratory rate, and WBC count parameters. Sepsis was defined as SIRS accompanied by a presumed or confirmed source of infection. Furthermore, this study also defined severe sepsis and septic shock as sepsis related to new or progressive multiple organ dysfunction and obligatory cardiovascular dysfunction, respectively. All age-related parameter cutoffs and definitions were conceived by expert consensus arising from the need for clear definitions for enrollment in research trials by the time. However, it soon became clear that the 2005 IPSCC criteria, while having high sensitivity, had low specificity (e.g., mild viral febrile illnesses could meet the SIRS criteria and be defined as sepsis), lacking the discriminative power to recognize those with increased risk of mortality, in addition to increasing the burden on health resources in the case of false positives (15). Since then, more recent studies on the epidemiology and recognition of pediatric sepsis have reinforced the need to review the criteria that define sepsis in children. The current adult consensus (Sepsis-3) cannot be automatically used in pediatrics (16), and, therefore, definitions specific to the child universe need to be reached. For these reasons, more than 15 years after its publication, 75% of studies on pediatric sepsis still adopt the 2005 IPSCC criteria for definition (12), despite its very low level of evidence (Table 1).

Questions regarding sepsis definition criteria also affect epidemiologic studies on pediatric sepsis, along with other factors as study design, population, and geographic region. Although sepsis epidemiology is the subject of 22% of the 50 most cited studies, 63% of these are retrospective cohorts using data from patient charts and case identification from administrative codes (Table 1). The inaccuracies and bias inherent to this study design are reflected in a low level of evidence and possibly contribute to discrepancies across prevalence rates. As an example, the 22nd and the 26th most cited publications depict different prevalence and mortality rates in US hospitals even using the same criteria (not the IPSCC in this case) and with both coming out in the same year (3, 17). Besides, only two of those eleven epidemiologic studies are not exclusively a US or a high-income country publication (1, 18), drawing attention to the paucity of data from Latin America, Asia, and Africa, suggesting that clinical research in these regions may still be based on isolated initiatives and large research networks and multicenter studies may not yet be consolidated (12).

### Sepsis Diagnosis

No targeted host immune response therapy has been proven to be effective in the treatment of sepsis so far. This means that success in sepsis treatment depends entirely on timely diagnosis and initiation of supportive measures. Therefore, identifying markers of disease severity could effectively reduce sepsis-associated mortality. One hope in this regard has been the search for ideal biomarkers. Biomarkers are “measurable and quantifiable biological parameters (e.g., specific enzyme concentration, specific hormone concentration, specific gene-phenotype distribution in a population, presence of biological substances) which serve as indices for health- and physiology-related assessments” (MeSH term, NLM/NCBI, USA). In SIRS, they can help differentiate the diagnosis of infection as opposed to other inflammatory syndromes, monitor response to treatment and predict outcomes in sepsis (19). The most studied biomarkers in the pediatric sepsis context have been C-reactive protein, procalcitonin, and lactate (20). Procalcitonin is more specific than C-reactive protein in the diagnosis of infection (37th most cited article) (19) and is related to the severity of organ failure and mortality in children with septic shock (32nd most cited) (21). High lactate levels are associated with mortality but have low sensitivity (7). Serum neutrophil gelatinase-associated lipocalin (NGAL) is a highly sensitive but non-specific predictor of acute kidney injury in septic shock (11th most cited) (22). Biomarkers can also be used in risk models to estimate mortality risk, as the Pediatric Sepsis Biomarker Risk Model (PERSEVERE) and Pediatric Sepsis Biomarker Risk Model-II (PERSEVERE-II), a revision of PERSEVERE which incorporates platelet count, enhancing its performance in patients with multiple organ failure, and also PERSEVERE-XP, which uses both protein and mRNA biomarkers to improve its performance and suggests that tumor protein 53-related cellular division, repair, and metabolism is involved in the biological pathways that drive poor outcome from septic shock (the original 2012 study is the 42nd most cited) (23–25). The 21st most cited article in the list, a study adapting and validating a pediatric score using Sepsis-3 criteria, also exemplify other risk models incorporating biomarkers together with clinical parameters (26). However, these are observational, prospective cohort studies, still classified as having a low level of evidence (Table 1).

On the other side, as pediatric sepsis lacks interventional studies (high level of evidence), it is difficult to prove or reject the effectiveness of the various therapies used so far. It is noteworthy that of the three studies with a high level of evidence on the list, two (among the 20 most cited) were randomized controlled trials of the use of activated protein C (6 and 16th most cited) (27, 28). In one of them (27), no differences were found in relation to the placebo group. The other (28) was an early century study in a specific population (severe meningococcal sepsis and purpura fulminans) seeking the best therapeutic dose. With these results and the accumulation of evidence to the contrary in the adult literature, this therapy was abandoned.

Current evidence suggest that we deal with a heterogenic population of septic patients, with specific pathophysiological mechanisms activated in certain subgroups. Identifying these prognostic mechanisms could mean placing a subset of patients to which selected therapies could be directed with an enhanced chance of successful response. These pathophysiological genetic sepsis profiles are called endotypes (20). In a *post-hoc*, secondary analysis, the application of PERSEVERE identified an endotype group of patients in which the use of corticosteroids was independently associated with a reduction in the risk of a complicated course (29). Strategies designed to sort out these groups of patients who are more likely to respond to specific therapies are called enrichment strategies. These sorting strategies can be based on the risk of an outcome, such as mortality (so-called prognostic enrichment) or specific pathophysiological patterns (so-called predictive enrichment) (25). These are the principles of precision medicine, which has been the focus of clinical trials designed to test sepsis-modifying therapies.

### Sepsis Management

While specific sepsis therapies are being studied and showing positive results in selected patients, protocolized sepsis supportive treatment as early antimicrobial therapy and fluid resuscitation have been proven effective in reducing morbidity and mortality in the general pediatric sepsis population (5, 17, 29, 34, 36, and 44th most cited studies, all cohorts or quality improvement studies) (30–35). In a tertiary care children's hospital in Philadelphia, USA, the use of a sepsis protocol in the emergency department was independently associated with a reduction in organ dysfunction compared to non-protocolized usual care (36). In a referral children's hospital in Rio de Janeiro, Brazil, dedicated to high complexity disease treatment, implementation of a sepsis protocol improved sepsis recognition and compliance with the first-h treatment bundle, and reduced the time interval to fluid resuscitation and antibiotics, as well as sepsis mortality (37). Similar results were found both in a study performed in 59 acute care hospitals in the New York State, USA (38) and another conducted in seven PICUs in a resource-limited setting (39), as well as in a systematic review and meta-analysis of fifty observational studies published previously (40). The benefits of protocolized sepsis treatment are well-established, highlighted by five publications of consensus definitions and clinical guidelines being within the 25 most cited publications—ranked 1st, 2nd, 4th, 12th, 24th (Table 1). The most recent Surviving Sepsis Campaign International Guidelines for the Management of Septic Shock and Sepsis-Associated Organ Dysfunction in Children recommend implementing a protocol for managing children with septic shock or another sepsis-associated organ dysfunction. However, most of their recommendations were weak due to the overall low quality of evidence, a trend also captured by our list (Table 1). Despite the clear benefits of protocolized care, these guidelines also consider the need for systematic screening to be tailored to the type of patients, resources, and procedures within each institution and recognize that variations across different settings should determine the practical application of the guidelines (41).

## Conclusion

In conclusion, the financial and health burden of sepsis is high worldwide, with significant disparities between high and LMIC. Consequently, sepsis research shows similar inequalities in different regions of the world. The most cited articles on pediatric sepsis in the 21st century address the challenge of establishing efficient sepsis criteria and the best protocol approach. More recent, highly cited studies focus on identifying sepsis severity, outcome biomarkers, and innovative specific treatment. However, most articles are still from observational studies with a low level of evidence. It is hoped that pediatric research may also grow toward more randomized controlled trials and more robust evidence in the coming years.

## Author Contributions

AP-B conceived the study. LM-R conducted the initial literature search and wrote the first draft of the manuscript. All authors analyzed the extracted data, contributed to the critical review, writing of the final version of the manuscript, and approving its final format.

## Funding

Funding for the presented work and publishing fees was provided by the Department of Pediatrics of D'Or Institute for Research and Education (IDOR).

## Conflict of Interest

The authors declare that the research was conducted in the absence of any commercial or financial relationships that could be construed as a potential conflict of interest.

## Publisher's Note

All claims expressed in this article are solely those of the authors and do not necessarily represent those of their affiliated organizations, or those of the publisher, the editors and the reviewers. Any product that may be evaluated in this article, or claim that may be made by its manufacturer, is not guaranteed or endorsed by the publisher.
